# Oral Exposure to *Chlorella sorokiniana* Detoxifies Deoxynivalenol, Ochratoxin A, and Fumonisin B1 In Vitro and In Vivo

**DOI:** 10.3390/toxins17070318

**Published:** 2025-06-23

**Authors:** Hiroki Yamaguchi, Mana Ando, Chiharu Ohira, Tensei Magami, Mao Kaneki, Kazutoshi Sugita, Taro Ogawa, Ayaka Nakashima, Tomoki Fukuyama

**Affiliations:** 1 School of Veterinary Medicine, Azabu University, 1-17-71 Fuchinobe, Chuo-ku, Sagamihara-shi 252-5201, Kanagawa, Japan; 2 Euglena Co., Ltd., 5-29-11 Shiba, Minato-ku, Tokyo 108-0014, Japan; 3 Center for Human and Animal Symbiosis Science, Azabu University, 1-17-71 Fuchinobe, Chuo-ku, Sagamihara-shi 252-5201, Kanagawa, Japan

**Keywords:** deoxynivalenol, mycotoxin, *Chlorella sorokiniana*, toxicokinetics

## Abstract

Mycotoxins are synthesized by various fungal species and are known to exert toxic effects on vertebrates and other animals, even at low concentrations. However, the current countermeasure for mycotoxin contamination is random inspection of samples prior to shipment. In this study, we focused on *Chlorella sorokiniana* (*CS*) from Ishigaki Island, Japan, and examined its ability to detoxify deoxynivalenol (DON), ochratoxin A (OTA), and fumonisin B1 (FB1) in vitro and in vivo. The binding of *CS* to DON, OTA, and FB1 was evaluated in vitro. The detoxification of *CS* was demonstrated by monitoring its concentrations in the plasma and urine samples of male ICR mice. Plasma and urine samples were collected 30 min, 2 h, and 24 h after an oral administration of 5 mg/kg mycotoxins and/or 500 mg/kg *CS*. *CS* bound to more than 80% and 40% of DON and OTA, respectively, whereas the binding of *CS* to FB1 was less than 10%. The concentrations of DON and OTA in plasma and urine samples were substantially reduced by *CS* co-administration, whereas CS did not affect FB1 absorption. The co-administration of *CS* substantially inhibited the systemic absorption of DON and OTA.

## 1. Introduction

Mycotoxins are low-molecular-weight natural products produced as secondary metabolites by filamentous fungi. These compounds are synthesized by various fungal species and are known to exert toxic effects on vertebrates and other animals, even at low concentrations. The diversity in chemical structures, biosynthetic origins, fungal sources, and toxicity profiles makes the definition of mycotoxins challenging and complicates their classification [1]. Fumonisins (FUM) and deoxynivalenol (DON) exhibit exceptionally high detection rates in many regions, particularly in the Middle East, North Africa, and China; the detection rate of ochratoxin A (OTA) is relatively low, although it is higher in Southeast Asia and Southern Africa [2]. In addition to the acute and high-dose toxicity of mycotoxins, chronic exposure to low mycotoxin concentrations has recently received increasing attention. We previously demonstrated that oral exposure to sub-chronic, low concentrations of DON or FUM substantially exacerbated local inflammation in mouse models and in in vitro immune cells [3,4,5,6]. Current countermeasures for mycotoxin contamination include monitoring the mycotoxin levels in feed ingredients, dietary manipulation, and on-farm management strategies [7]. Countermeasures against the toxicity or absorption of mycotoxins are under-explored. Finding therapeutic agents against mycotoxin-induced toxicity is not realistic because of their diverse toxicological properties and multiplex influences. In the present study, we focused on a nutritional agent that prevents the absorption of mycotoxins during oral ingestion.

Various mycotoxin-binding agents have been investigated thus far, and some have been put to practical use in commercial animal production. Phyllosilicates, tectosilicates, activated coating, montmorillonite clays, and bentonite as inorganic agents and polysaccharide beta glucans, yeast cell walls, live yeast *Saccharomyces cerevisiae,* and plant extracts as organics have previously been investigated. Hojati et al. [8] evaluated bentonite clay, a yeast cell wall product, and an activated charcoal product in vitro to verify their ability to bind aflatoxin B1 (AFB1). Bentonites showed high adsorption ability, binding to more than 70% of the available AFB1. The yeast cell wall showed moderate adsorption ability, binding approximately 47% of the available AFB1. The adsorption ability of activated carbon was substantially lower than that of other binders [8]. These binders were demonstrated to be able to bind other mycotoxins, including OTA and FUM; however, the binding properties of each binder differed depending on the type of mycotoxin and the condition of each agent [9]. Moreover, the use of these binders should be carefully considered based on their influence on the absorption of other nutrients and potential adverse effects.

In terms of some natural materials, *Chlorella* spp., which are freshwater photosynthetic microalgae, have gained increasing attention because of their high content of protein, vitamins, and minerals, as well as their extensive use in food and dietary supplements [10]. Given their favorable safety profile and nutritional benefits, *Chlorella* spp. have been regarded as promising candidates for use in functional foods with detoxifying potential, especially for long-term consumption. Numerous studies have suggested that *Chlorella* spp. exhibits detoxifying activities against various environmental pollutants. For example, a clinical study involving 44 pregnant women in Japan showed that those who consumed *Chlorella pyrenoidosa* during gestation (*n* = 23) had approximately 30% lower concentrations of total dioxin toxic equivalents in their breast milk than a control group [11]. In animal models, rats fed a diet containing 10% *Chlorella* exhibited increased fecal excretion of polychlorinated dibenzo-p-dioxins and polychlorinated dibenzofurans, with excretion levels elevated by 0.3–3.4-fold and 0.5–2.5-fold, respectively, compared to controls (most comparisons, *p* < 0.05) [12]. The co-administration of *Chlorella vulgaris* and aflatoxin-contaminated feed has been shown to substantially reduce tissue residue levels of aflatoxins in quails [13]. Although several studies have suggested the potential detoxifying activity of *Chlorella* spp. against multiple mycotoxins, to the best of our knowledge, no prior investigations have specifically evaluated the mycotoxin-binding capacity of CS, a commercially available strain cultivated in Japan.

Ishiguro et al. [14] demonstrated that the cell wall membrane fraction of CS from Ishigaki Island has antitumor immunity and inhibits colon carcinoma growth in mice. Several studies also indicated that CS significantly enhanced immune responses [15]. On the other hand, its detoxification potential has not been examined so far. To find a novel mycotoxin binder without potential adverse effects, we focused on *Chlorella sorokiniana* (CS) from Ishigaki Island in Japan and examined its detoxification of the highly prevalent mycotoxins DON, OTA, and fumonisin B1 (FB1) in vitro and in vivo.

## 2. Results

**Figure 1:**
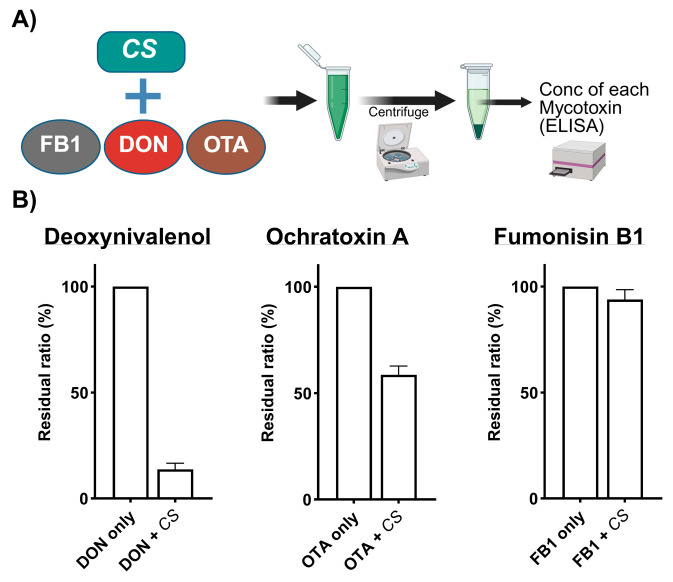
Experimental protocol to evaluate the binding property of *Chlorella sorokiniana* (CS) to mycotoxins (DON, OTA, FB1). (**A**) The concentration of each mycotoxin in the supernatant was evaluated by a specific enzyme-linked immunosorbent assay (ELISA). (**B**) The residual ratio (%) was calculated as the residual to original mycotoxin concentration DON = deoxynivalenol; ochratoxin A = OTA; fumonisin B1 = FB1.

The binding of CS to DON and OTA was monitored in vitro (Figure 1A). More than 80% and 40% of DON and OTA, respectively, was bound, whereas the binding property of CS to FB1 was less than 10% in in vitro experiments (Figure 1B).

**Figure 2:**
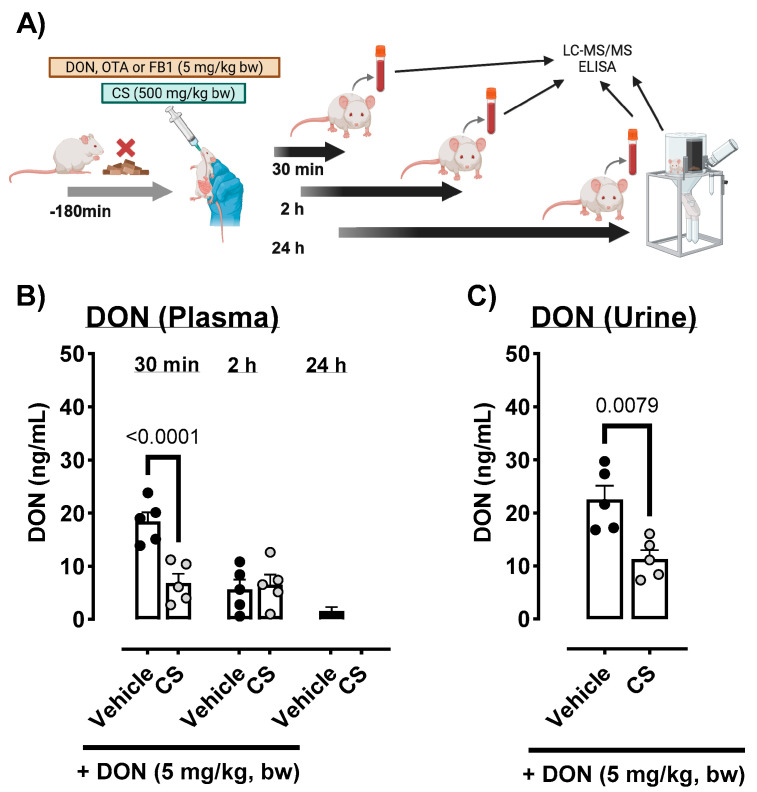
Experimental protocol of the toxicokinetic study in ICR mice (**A**) The protocol was administered after feed withdrawal. (**B**) Concentrations of DON (+AcOH-1) in the plasma 30 min to 24 h were evaluated using liquid chromatography with tandem mass spectrometry (LC-MS/MS). (**C**) DON levels in accumulated urine samples were also measured using ELISA. Results are presented as the mean (ng/mL) ± standard error of mean (*n* = 5 per group). *p* < 0.05 (unpaired *t*-test) vs. the vehicle-only control group; CS, *Chlorella sorokiniana,* DON = deoxynivalenol; LC/MS/MS = liquid chromatography/mass spectrometry; ochratoxin A = OTA; fumonisin B1 = FB1.

Next, the absorption of each mycotoxin was confirmed in male ICR mice by oral administration of 5 mg/kg body weight (BW) DON, OTA, or FB1 with or without 500 mg/kg BW CS (Figure 2A). The influence of oral CS exposure on the absorption and metabolism of each mycotoxin was examined by measuring the plasma and urine concentrations of each mycotoxin. The concentration of DON (DONAcOH) in the plasma 30 min after oral administration was substantially lower after the administration of CS (Figure 2B and Supplemental Figure S1). There was no difference in plasma concentrations between the vehicle control and CS treatment groups 2 h and 24 h after oral administration (Figure 2B and Supplemental Figure S1). DON levels in urine samples were substantially decreased by CS treatment (Figure 2C).

**Figure 3:**
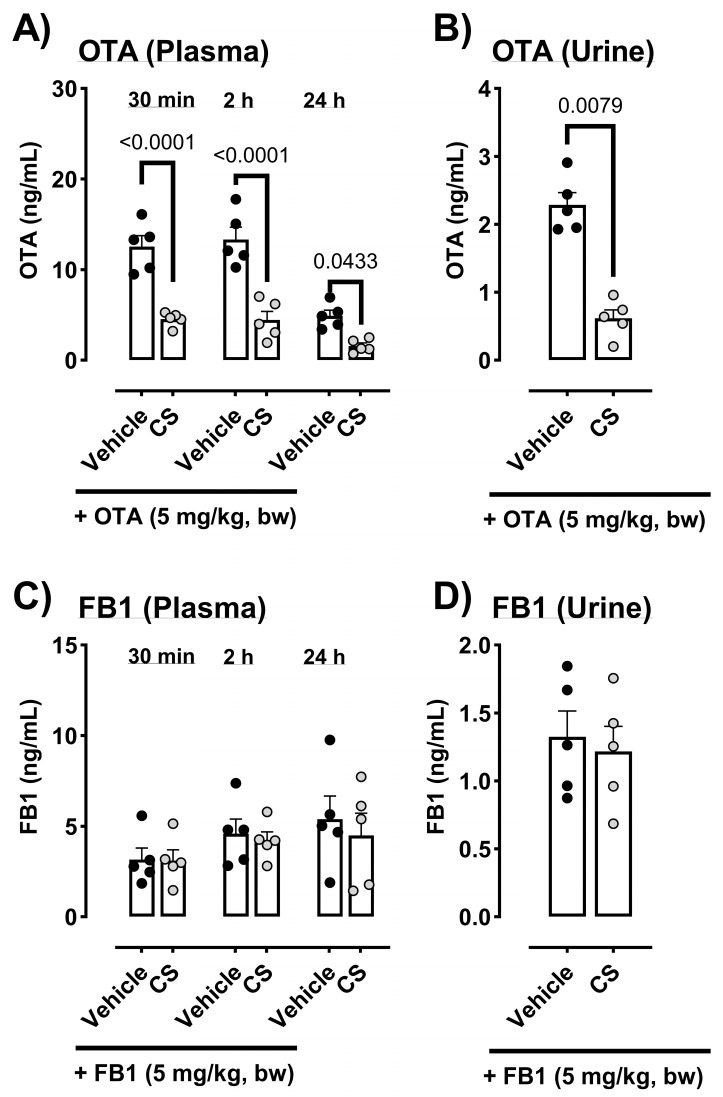
(**A**) Concentrations of ochratoxin A (OTA) in the plasma 30 min to 24 h after oral administration were markedly decreased by CS treatment. (**B**) A marked reduction in OTA in the urine samples 24 h after oral administration was observed in the CS treatment compared to that of OTA-only-treated mice. The FB1 concentration in (**C**) plasma and (**D**) urine samples was not influenced by CS treatment. Results are presented as the mean (ng/mL) ± standard error of mean (*n* = 5 per group); *p* < 0.05 (unpaired *t*-test) vs. the vehicle-only control group; CS, *Chlorella sorokiniana* DON = deoxynivalenol; ochratoxin A = OTA; fumonisin B1 = FB1.

In vivo studies were performed using OTA and FB1 in the same settings as for DON. The concentration of OTA in the plasma was substantially decreased by the administration of CS at all sampling time points (30 min, 2 h, and 24 h after oral administration) (Figure 3A). A marked reduction in OTA in urine samples collected 24 h after oral administration was also observed in CS-treated mice compared to that in OTA-only-treated mice (Figure 3B). No marked effect on FB1 concentration in the plasma or urine samples was observed after additional CS treatment (Figure 3C,D).

## 3. Discussion

DON is a mycotoxin primarily found in cereals and grain-derived foods, and its adverse effects on the immune system and its role in inducing inflammation have been previously demonstrated [3,6,16]. OTA, primarily found in dietary products, is one of the most important mycotoxins due to its widespread occurrence and pronounced toxicological effects [17,18]. Exposure to OTA has been associated with many diseases and toxic responses, including nephrotoxicity, blood–brain barrier damage, developmental toxicity, neurotoxicity, mutagenicity, and hepatotoxicity [19,20,21]. It is carcinogenic to poultry and rodents and is classified as a Group 2B carcinogen in humans. A recent epidemiological study detected fumonisin in 60% of 74,821 samples collected from 100 countries between 2008 and 2017, with the highest median concentration at 723 µg/kg [22]. Previous reports demonstrated that exposure to fumonisin causes encephalomalacia in horses, pulmonary edema in pigs, neural tube defects in human fetuses, esophageal cancer in humans, and the induction of several allergies in a mouse model. These effects are potentially caused by fumonisin’s inhibition of the sphingolipid biosynthesis pathway [23,24,25]. To develop comprehensive treatment strategies for the contamination of these mycotoxins, we focused on CS and examined its binding properties to DON, OTA, and FB1 in vitro and the inhibitory pattern of absorption and metabolism in vivo.

When integrated with previous findings on the detoxification capacity of *Chlorella* spp. toward environmental pollutants, such as dioxins and aflatoxins, the present results further suggest that CS exhibits selective adsorptive properties toward mycotoxins and related toxins with moderate molecular weights. Specifically, CS showed strong binding and absorption-suppressive effects against DON (MW: 296.3 Da) and OTA (MW: 403.8 Da), which are comparable in size to aflatoxin B1 (MW: 312.3 Da) and 2,3,7,8-tetrachlorodibenzo-p-dioxin (MW: 321.9 Da), both of which have previously been shown to be effectively bound or excreted in the presence of *Chlorella*. In contrast, no appreciable adsorption or in vivo absorption suppression was observed for FB1, which is a substantially larger molecule with a molecular weight exceeding 700 Da. These findings imply that the binding efficacy of *Chlorella* spp. may be structurally constrained, with optimal interactions occurring primarily for compounds within a molecular weight window of 300–400 Da.

Structurally, *Chlorella* spp. possess thick and highly resilient cell walls composed of complex polysaccharides, including cellulose-, dextrin-, and chitin-like substances. These unique structural components may contribute to their selective binding capabilities and limited bioavailability and are likely key determinants of their adsorptive interactions with environmental toxins [26]. This complex, cross-linked, high-molecular-weight network structure is believed to provide the physical basis for the adsorptive capacity of *Chlorella*, enabling them to bind a wide variety of toxic substances. The cell wall is particularly rich in polysaccharides, proteins, and glycoproteins that interact with heavy metals and other toxic compounds. Through these interactions, *Chlorella* spp. are thought to effectively reduce the bioavailability of harmful substances, promote their elimination from the body, and mitigate their toxic effects [27].

In the present study, CS demonstrated a direct binding capacity when mixed in vitro with DON and OTA, indicating its ability to adsorb these mycotoxins onto the cell surface. When comparing the plasma absorption profiles of OTA and DON in this study, the in vivo results did not appear to fully correlate with the in vitro binding data. Although OTA exhibited a relatively low adsorptive capacity in vitro, it showed a pronounced inhibitory effect on systemic absorption in vivo, with substantially reduced plasma concentrations observed at both 2 and 24 h after dosing following co-administration with CS. This anomaly may be attributed to the pharmacokinetics of OTA in rodents, particularly its well-characterized enterohepatic circulation [28]. OTA, which undergoes enterohepatic recirculation, may be re-exposed to CS in the intestinal lumen, facilitating additional binding and enhancing its inhibitory effect on reabsorption. In contrast, the inhibitory effect of CS on DON absorption was limited. No statistically significant reduction in plasma concentrations was observed for DON (DONAcOH) at 2 or 24 h after dosing. This may be explained by the pharmacokinetic characteristics of DON, as most parent compounds are rapidly eliminated via urinary excretion in rodents [29].

Taken together, these findings suggest that lipophilic toxicants with molecular weights of 300–400 Da are more likely to undergo biliary excretion, followed by intestinal reabsorption. When CS is administered concurrently, these compounds may be adsorbed onto the surface of the CS cell walls in the intestinal lumen, thereby suppressing their reabsorption and enhancing their elimination from the body. However, for compounds that are primarily excreted via the urinary route, the inhibitory effect of CS on their absorption appears to be limited, unless there is simultaneous exposure to CS within the gastrointestinal tract. Our findings that CS did not affect the absorption of FB1 is another limitation of CS. The mechanism of how to bind CS to DON and OTA is a remaining question in the future, and the reason for it not working with FB1 should be also cleared up in future research. On the other hand, compared to the other binders to mycotoxins, the cell wall membrane fraction of CS from Ishigaki Island has nutritional advantages, including antitumor and immune-enhancing properties [14,15]. Our future studies will focus on the direct or indirect anti-inflammatory properties of CS related to exposure to DON or OTA.

## 4. Conclusions

The current study provides evidence that CS specifically binds to DON and OTA and that supplemental treatment with CS in food may inhibit the absorption and metabolism of DON and OTA in vivo. However, the effects of CS on FB1 were not observed in vitro or in vivo; thus, the binding properties of CS may differ depending on the type and concentration of mycotoxins. We are currently performing subsequent studies to focus on the inhibitory effects of CS treatment on DON- or OTA-specific diseases such as allergy and kidney failure.

## 5. Materials and Methods

### 5.1. Binding Property of CS to DON, OTA, and FB1

DON, OTA, and FB1 standards were obtained from FUJIFILM Wako Pure Chemical Corporation (Osaka, Japan). Mycotoxins (1 mg) and CS (100 mg) were mixed in 1 mL of ultrapure deionized water (DON and FB1) or corn oil (OTA) and incubated for 10 min at room temperature. Heated-air-dried CS powder was obtained from Euglena Co., Ltd. (Tokyo, Japan). The CS powder (100 g) used in this study contained 4.3 g of water, 69.58 g of protein, 5.1 g of ash, 98 mg of sodium, 27.3 mg of iron, and 2.91 g of total chlorophyll. After 10 min of incubation, the mixed solution was centrifuged (200× *g*) for 5 min, and each mycotoxin concentration was determined using an enzyme-linked immunosorbent assay (ELISA) according to the supplier’s instructions (DON, Nittobo Medical Co., Ltd., Tokyo, Japan; OTA, Meizheng Bio-Tech, Shandong Province, China; FB1, Elabscience Bionovation Inc., Houston, TX, USA). The detection limits of the DON, OTA, and FB1 kits were 4.12, 0.05, and 1 ng/mL, respectively. The residual ratio (%) was calculated using the residual mycotoxin and the original mycotoxin concentrations in the supernatant (Figure 1A).

### 5.2. Mycotoxin Concentrations in Plasma and Urine Samples After Oral Exposure to 5 mg/kg Body Weight of DON, OTA, or FB1 in Male ICR Mice

Seven-week-old male ICR mice were obtained from Japan SLC, Inc. (Shizuoka, Japan). The mice were housed under a 12 h light/dark cycle at 22 ± 3 °C and humidity of 50 ± 20%. Feed and water were provided ad libitum. All procedures were conducted in accordance with the Animal Care and Use Program of Azabu University (approval no. 220316-46). The experimental protocol is shown in Figure 2A. Mice were fasted for 3 h prior to oral administration of mycotoxins with or without CS. DON was dissolved in deionized water and OTA and FB1 were dissolved in corn oil (Fujifilm Wako Pure Chemical Corporation, Osaka, Japan) at 5 mg/kg body weight (BW). In the CS-treated group, 500 mg/kg BW CS in deionized water was administered to mice using an oral gavage. Mice were euthanized under isoflurane anesthesia 30 min, 2 h, and 24 h after oral administration, and plasma and urine (24 h only) samples were collected for further analysis.

For DON analysis, a portion of the plasma was subjected to protein precipitation for LC-MS/MS analysis [30]. Briefly, 400 µL of ice-cold ethanol and 50 µL of 20% trichloroacetic acid were added to a sample of 250 µL of plasma. Proteins were precipitated by centrifugation at 6200× *g* for 15 min at 4 °C several times. A mixture of 20% trichloroacetic acid, saline, and ethanol at a ratio of 1:5:8 was prepared, and the supernatant from the plasma sample was diluted with this solution. The following conditions were used: column: ZORBAX Eclipse XDB C-18 RRHD Solvent Sa vor (150 mm × 2.1 i. d. mm, 1.9 μm, Agilent Technologies, Santa Clara, CA, USA), eluent A: water/acetic acid (99.9/0.1, *v*/*v*) containing 0.5 mmol/L ammonium acetate, eluent B: acetonitrile/acetic acid (99.9/0.1, *v*/*v*), with stepwise gradient for 13.8 min (0–1 min: hold 12% B; 1–6.5 min: from 12% to 68% B; 6.5–9 min: from 68% to 85% B; 9–10 min: hold 85% B; 10–10.1 min: from 85% to 12% B; 10.1–13.8 min: hold 12% B to equilibrate); flow rate: 0.3 mL/min; injection volume: 3 μL; oven temperature: 40 °C; ionization: negative ion mode electrospray. A serum sample (without any pretreatment) was used for OTA and FB1 analysis. The detection limit was 0.1 ng/mL. ELISA kits were used for the quantification of OTA and FB1, as described in Section 5.1.

### 5.3. Statistical Analysis

All data are expressed as the mean ± standard error of the mean (SEM). Analysis of variance (ANOVA) was conducted (Bartlett’s test and the Brown–Forsythe test), followed by Šídák’s multiple comparison test or an unpaired *t*-test with Welch’s correction to determine significant differences between the values of multiple or two groups. Statistical significance was set at 5%. Data were analyzed using GraphPad Prism 10 (GraphPad Software, San Diego, CA, USA). 

## Data Availability

The original contributions of this study are included in this article. Further inquiries can be directed to the corresponding author.

## Supplementary Materials

The following supporting information can be downloaded at: https://www.mdpi.com/article/10.3390/toxins17070318/s1, Figure S1: Concentrations of DON in analyzed by several detective ions, (A) + AcOH-1, (B) + AcOH-2, (C) + AcOH-3, (D) + AcOH-4, and (E) + AcOH-5 in the plasma 30 min to 24 h were evaluated using liquid chromatography with tandem mass spectrometry (LC-MS/MS). Results are presented as the mean (ng/mL) ± standard error of mean (n = 5 per group). *p* < 0.05 (unpaired *t*-test) vs. the vehicle-only control group; CS, *Chlorella sorokiniana,* DON = deoxynivalenol.

## Abbreviations

The following abbreviations are used in this manuscript:

DON	deoxynivalenol
ELISA	enzyme-linked immunosorbent assay
FB1	fumonisin B1
LC/MS/MS	Liquid Chromatography/Mass Spectrometry
OTA	ochratoxin A
SEM	standard error of the mean
