# Supplementary material for: Oral Exposure to Chlorella sorokiniana Detoxifies Deoxynivalenol, Ochratoxin A, and Fumonisin B1 In Vitro and In Vivo
Source: Toxins (Basel). 2025 Jun 23;17(7):318. doi: 10.3390/toxins17070318 (PMC12300608; doi:10.3390/toxins17070318)
Supplement: Supplementary file 1 [file toxins-17-00318-s001.zip › toxins-3682374-supplementary.pdf]

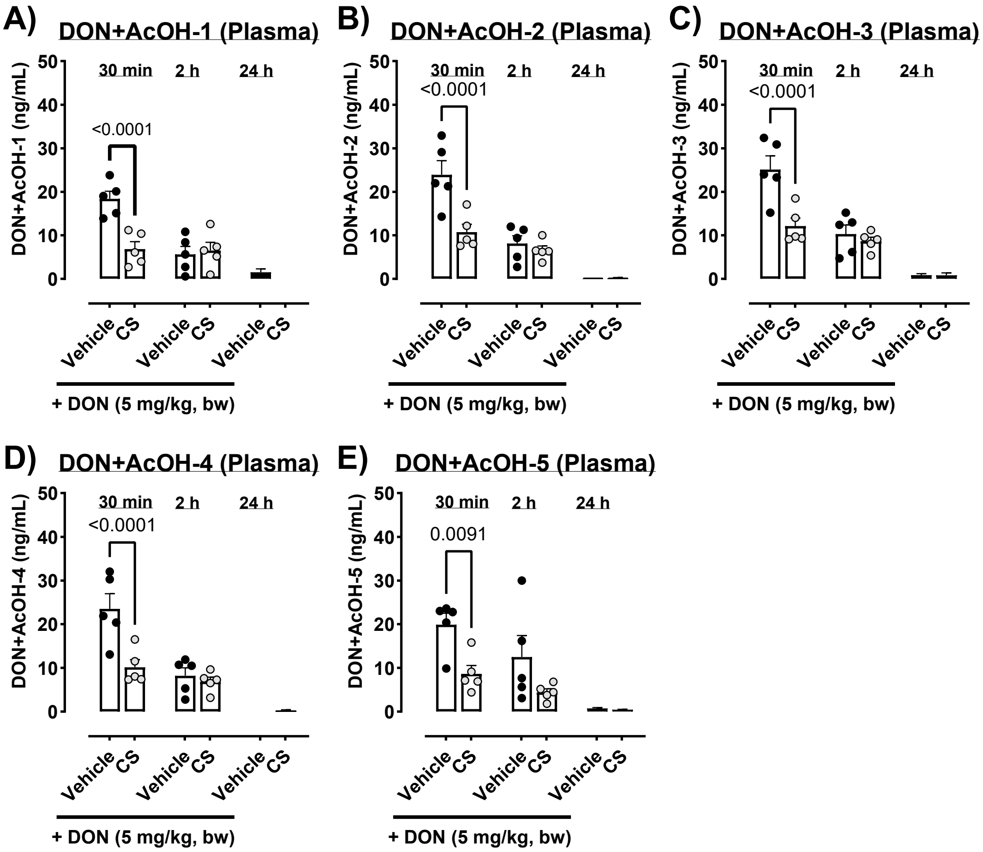

Supplemental Figure S1. Concentrations of DON in analyzed by several detective ions, (A) + AcOH-1, (B) + AcOH-2, (C) + AcOH-3, (D) + AcOH-4, and (E) + AcOH-5 in the plasma 30 min to 24 h were evaluated using liquid chromatography with tandem mass spectrometry (LC-MS/MS). Results are presented as the mean (ng/mL) ± standard error of mean (n = 5 per group). P <0.05 (unpaired t-test) vs. the vehicle-only control group; CS, *Chlorella sorokiniana*, DON = deoxynivalenol

 https://doi.org/10.3390/toxins17070318
